# Infant rhesus macaques as a non-human primate model of *Bordetella pertussis* infection

**DOI:** 10.1186/s12879-021-06090-y

**Published:** 2021-05-03

**Authors:** Wenwen Jiang, Chen Wei, Dachao Mou, Weilun Zuo, Jiangli Liang, Xiao Ma, Lichan Wang, Na Gao, Qin Gu, Peng Luo, Yan Ma, Jingyan Li, Shuyuan Liu, Li Shi, Mingbo Sun

**Affiliations:** 1 Institute of Medical Biology, Chinese Academy of Medical Science & Peking Union Medical College, Kunming, 650118 Yunnan China; 2 Yunnan Key Laboratory of Vaccine Research and Development on Severe Infectious Diseases, Kunming, 650118 Yunnan China; 3 Department of Diphtheria, Tetanus and Pertussis Vaccine and Toxins, National Institute for Food and Drug Control, Beijing, China

**Keywords:** *Bordetella pertussis*, Rhesus macaques, Infection, Transmission

## Abstract

**Background:**

The prevalent resurgence of pertussis has recently become a critical public health problem worldwide. To understand pertussis pathogenesis and the host response to both the pathogen and vaccines, a suitable pertussis animal model, particularly a non-human primate model, is necessary. Recently, a non-human primate pertussis model was successfully established with baboons. Rhesus macaques have been shown to be ideal animal models for several infectious diseases, but a model of infectious pertussis has not been established in these organisms. Studies on rhesus macaque models of pertussis were performed in the 1920s–1930s, but limited experimental details are available. Recent monkey pertussis models have not been successful because the typical clinical symptoms and transmission have not been achieved.

**Methods:**

In the present study, infant rhesus macaques were challenged with *Bordetella pertussis* (*B.p)* using an aerosol method to evaluate the feasibility of this system as an animal model of pertussis.

**Results:**

Upon aerosol infection, monkeys infected with the recently clinically isolated *B.p* strain 2016-CY-41 developed the typical whooping cough, leukocytosis, bacteria-positive nasopharyngeal wash (NPW), and interanimal transmission of pertussis. Both systemic and mucosal humoral responses were induced by *B.p*.

**Conclusion:**

These results demonstrate that a model of pertussis was successfully established in infant rhesus macaques. This model provides a valuable platform for research on pertussis pathogenesis and evaluation of vaccine candidates.

**Supplementary Information:**

The online version contains supplementary material available at 10.1186/s12879-021-06090-y.

## Background

Pertussis is an acute respiratory disease caused mostly by the gram-negative bacterium *Bordetella pertussis* (*B.p)*. The basic illness is non-inflammatory in nature and occurs without significant fever. The disease is characterized by non-productive paroxysmal coughs followed by periods of total respiratory normalcy, which makes it different from all other infectious cough illnesses [1]. Severe respiratory failure is complicated by pulmonary hypertension, which may cause death, especially in infants [2]. Pertussis is transmitted directly from human to human, mostly via aerosolized respiratory droplets. Pertussis is a preventable disease, and its incidence decreased notably after vaccine immunization began. However, it has experienced a resurgence in several countries, even in countries with nearly universal vaccine coverage in the last 20 years [3–5]. A deeper understanding of the mechanism of pertussis pathogenesis and the host response to both the pathogen and the vaccines is urgently needed to enable this important public health concern to be faced. Therefore, it’s urgent to develop suitable animal models for pertussis.

To establish animal models for pertussis, several studies have been carried out in mouse, rat, rabbit, and piglet models of pertussis [6, 7]. Unfortunately, these models have not been able to reproduce the full clinical spectrum observed in humans [8, 9]. In studies using non-human primate (NHP) models, a baboon model has been successfully established [10]. Low-grade fever, paroxysmal coughing, leukocytosis, a long-lived anti-pertussis toxin (PT) antibody response, protection against subsequent challenge, and transmission have been achieved in this baboon model, which makes the model crucial for studies on the pertussis pathogenic mechanism as well as for the development of new vaccines and therapeutics [11]. Another NHP, the rhesus macaque, has been evaluated for use as a pertussis model since 1929, but none of the studies have been able to completely replicate the human clinical disease [12–14]. In contrast, 2 studies using *Macaca (M.) cyclopis* have investigated the similarity of the disease in these organisms to the human pertussis clinical syndrome [15, 16]. Baboons and macaques are Old World monkeys that were separated approximately 10 million years ago, and rhesus macaques are closely related to *M. cyclopis.* Several disease models have been established using macaques and/or baboons. While baboon models suffer from limited availability, high housing costs, and a lack of suitable reagents for use in these monkeys, rhesus macaques are more readily available and have low housing costs, and suitable reagents are available [17].

Therefore, we infected rhesus macaques with *B.p* via aerosol challenge. We investigated clinical symptoms, including leukocytosis, coughing, and nasopharyngeal colonization; analysed the humoral and mucosal immune response and cytokine levels; and performed a transmission test to evaluate the suitability of infant rhesus macaques as a potential alternative NHP model for pertussis.

## Methods

### Animals

The infant rhesus macaques used in this study (5–6 months of age) were obtained from the Institute of Medical Biology, Chinese Academy of Medical Sciences (IMBCAMS). The study protocol was approved (DWSP201809002) by the Committee on Ethics of the IMBCAMS, and the study was conducted in strict accordance with the Guidelines for the Care and Use of Laboratory Animals published by the National Research Council of the National Academies and the Guidance for Experimental Animal Welfare and Ethical Treatment published by the Ministry of Science and Technology of the People’s Republic of China (2006). During the study periods, the monkeys were maintained at Animal Biosafety Level 2, housed individually in cages in a climate-controlled room (temperature of 18–25 °C and humidity 30–70%) with a 12 h light/dark cycle, given chow and fruits in strictly accordance with the animal welfare requirements and allowed free access to water. After the experiment, the monkeys were confirmed to have completely recovered from *B.p* infection and were returned to the Monkey Mountain of IMBCAMS, where they were allowed to live until they died naturally.

### Bacterial strains and media

The *B.p* strain 2016-CY-41 used in this study was recently isolated from a patient in China and was obtained from the National Institutes for Food and Drug Control (Beijing, China). The polymorphisms in the PT promoter (ptxP), PT subunit 1 (ptxA), pertactin (prn), fimbrial (fim)2 and fim3 were assessed by DNA sequencing. The genotype of 2016-CY-41 was ptxP1/ptxA1/prn1/fim2–1/fim3–1. For *B.p* infection experiments, bacteria were grown on Bordet-Gengou agar (B-G) plates (BG, Hopebio, CHN) containing 20% defibrinated sheep blood (Nanjinglezhen, CHN) for 48 to 72 h at 37 °C. Colonies from fresh B-G plates were resuspended in isotonic saline, diluted to a concentration of 10^11^ CFU/mL using a turbidimetric method, and used within 2 h of preparation. For culture of nasopharyngeal wash (NPW) bacteria, Regan-Lowe plates prepared from Regan-Lowe charcoal agar base with 10% defibrinated sheep blood and 40 μg/mL cephalexin (Oxoid, US) were used.

### Infection and transmission in rhesus macaques

Seven healthy male rhesus macaques, aged 5 to 6 months and weighing 1.2–1.8 kg, were obtained from the IMBCAMS (Animal Licence No. SCXK (Dian) K2015–0004). Before the experiment started, immunoglobulin G (IgG) against filamentous haemagglutinin (FHA) was detected to confirm that the monkeys were negative for infections of *B.p*, *B. parapertussis,* and *B. bronchiseptica*. Then, the seven animals were randomly assigned to two groups (Table 1). Group 1, containing 5 macaques, was challenged with strain 2016-CY-41 via aerosol exposure using an aerosolization apparatus designed by our laboratory and produced by Lanfang Honlan Equipment Co (Additional file 1). The apparatus was composed of a rectangular Plexiglas chamber with a removable lid (40 cm × 60 cm × 40 cm), a pump and a medical nebulizer (average atomization rate: ≥ 0.15 mL/min, working pressure: 60–150 kPa, normal working temperature: 10–40 °C). The pump was connected to the inlet side of the nebulizer to deliver a *B.p* suspension for atomization. The outlet side of the nebulizer was connected to two inlet ports of the challenge chamber to deliver atomized *B.p* to the interior of the chamber. An outlet tube with an air filter was connected to the challenge chamber to remove air. An air sampling port was embedded in the middle of the challenge chamber to monitor the actual concentration of aerosolized *B.p* inside the chamber. Animals were infected via the challenge chamber for 60 min. Within the 60 min period, the air sample was removed from the sampling port every 10 min for assessment of the concentration of *B.p* inside the chamber.

**Table 1:** Experimental grouping for *Bordetella pertussis* infection in rhesus macaques

Group	Monkey ID	Sex	Age (month)	Weight (kg)	*B.p* Strain	Infection route
Group 1	18,089	M	6	1.5	2016-CY-41	Aerosol challenge
	18,105	M	6	1.8	2016-CY-41	Aerosol challenge
	18,043	M	5	1.1	2016-CY-41	Aerosol challenge
	18,053	M	5	1.2	2016-CY-41	Aerosol challenge
	18,093	M	6	1.5	2016-CY-41	Aerosol challenge
Group 2	18,073	M	6	1.5	2016-CY-41	Transmission
	18,107	M	5	1.2	2016-CY-41	Transmission

At 2 days post infection (dpi), 1 challenged macaque was cohoused with 1 naive animal in one cage, and the animals were separated after 4 days to investigate transmission. The 2 macaques that were cohoused with 2016-CY-41-challenged animals formed group 2.

### Animal evaluation and sample collection

**Fig. 1:**
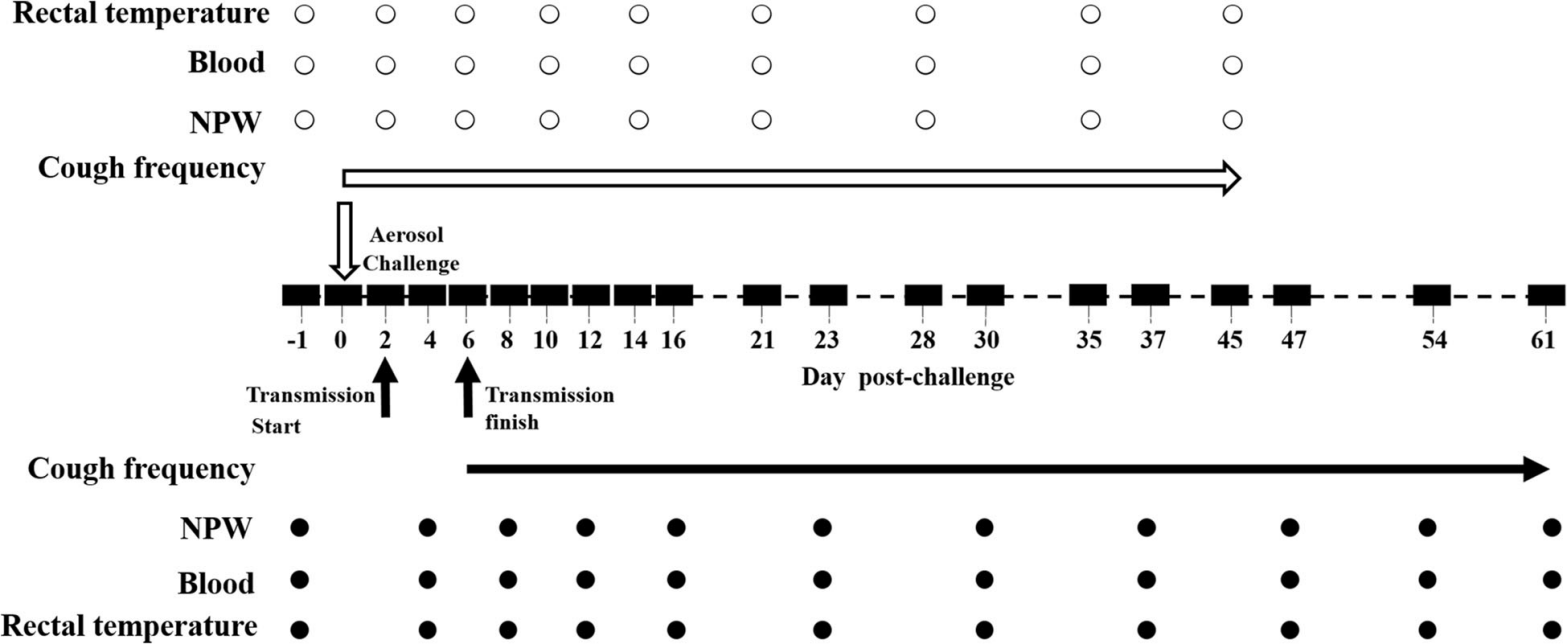
Timeline of *B.p* aerosol challenge and sample collection from rhesus macaques. Infant rhesus macaques were infected with *B.p* by aerosol challenge on day 0 (empty arrow). A naive animal and a challenged animal were cohoused in one cage at 2 dpi for transmission of the infection (solid arrow). The circles indicate detection time points (empty for aerosol-challenged animals; solid for transmission animals). Coughing frequency was monitored every day

A schematic of the specimen collection timeline is displayed in Fig. 1. Total white blood cell (WBC) counts were measured by blood cell counting. Coughing frequency was monitored with a recording device. The data were reviewed, and the numbers of coughs during four 30-min periods each day (7:00–7:30 a.m., 10:00–10:30 a.m., 2:00–2:30 p.m., and 8:00–8:30 p.m.) were calculated. The average number of coughs per hour for each day was calculated as the mean for all four observation periods for all animals in each group. For NPW collection, animals were anaesthetized using ketamine hydrochloride (10 mg/kg). A piece of tubing approximately 15 cm in length and 0.6 mm in diameter was slowly inserted into the back of the nostril. A syringe connected to the end of the tubing was used to slowly inject 1 mL of PBS into the nostril, and fluid was collected in a sterile dry container. This process was repeated for the other nostril, and fluid was collected into the same container as that used for the first nostril. The NPW was serially diluted in saline and plated on Regan-Lowe plates. The number of CFUs was calculated after 4–5 days of incubation at 37 °C. The *B.p* colonies were identified by examining colony morphology and haemolysis on Regan-Lowe plates and by polymerase chain reaction (PCR) amplification of IS481, a genomic insertion site that is specific for *B.p* [18].

### Detection of antibodies in serum and NPW

Serum and NPW were collected, and anti-PT, anti-FHA, anti-PRN and/or anti-adenylate cyclase toxin (ACT) IgG and/or IgA levels were measured using an enzyme-linked immunosorbent assay (ELISA). Microplates (96-well) were coated with the antigens PT, FHA, PRN, and/or ACT at 3 μg/mL and incubated at 4 °C overnight. Then, the plates were blocked with 3% (w/v) bovine serum albumin (BSA, Amresco, A0332) in phosphate-buffered saline (PBS) at 37 °C for 2 h. Diluted serum or NPW was added to each microplate and incubated at 37 °C for 1 h. After washing, horseradish peroxidase (HRP)-labelled sheep anti-monkey IgG (Invitrogen, USA, PA1–84631) or anti-human IgA (Jackson ImmunoResearch Laboratories, USA, 109–036-011) was added to the microplate, and the plate was incubated at 37 °C for 1 h. All of the ELISA plates were developed using tetramethylbenzidine (TMB; Solarbio, CHN, PR1200) to generate a colorimetric reaction, and the reaction was terminated with 2 mol/L H_2_SO_4_. For each set of ELISA plates subjected to IgG detection, a pertussis antiserum WHO international standard was used as a reference (NIBSC code: 06/140). For assessment of anti-ACT IgG in serum and antibody responses in NPW, a blank sample was included on each plate, and an optical density (OD) values ≥2.1-fold that of the blank sample was set as the cut-off value (all the antigens were from the Department of DTP Vaccine and Toxin, National Institute for Food and Drug Control, China). The results are presented as geometric mean concentrations (GMCs) or geometric mean titres (GMTs) and their 95% confidence intervals (CIs).

### Measurement of cytokines

Serum concentrations of interleukin (IL)-1β, IL-4, IL-6, IL-8, IL-10, IL-12/23p40, IL-13, IL-17A, interferon (IFN)-γ, and tumour necrosis factor (TNF)-α were detected by the Luminex technique with a MILLIPLEX NHP Magnetic Bead Panel (Merck Millipore, US) according to the manufacturer’s instructions. An unpaired t-test was used to test for differences between the pre-challenge cytokine production and the peak cytokine production during the post-challenge period (2/4, 6/8, 10/12, 14/16, 21/23, and 28/30 dpi; the latter is for the transmission group, as indicated in Fig. 1) for each animal due to the highly variable starting concentrations between animals and the variability of the peak response for each cytokine post infection.

### Statistics

The data were graphed and analysed using GraphPad Prism version 7.0 (GraphPad Software, Inc.). The data are presented as the means ± standard errors of the means or as the GMCs/GMTs and their 95% CIs. Unpaired Student’s t-test was utilized to assess statistical significance.

## Results

### Clinical signs in infant rhesus macaques after *B.p* infection

**Fig. 2:**
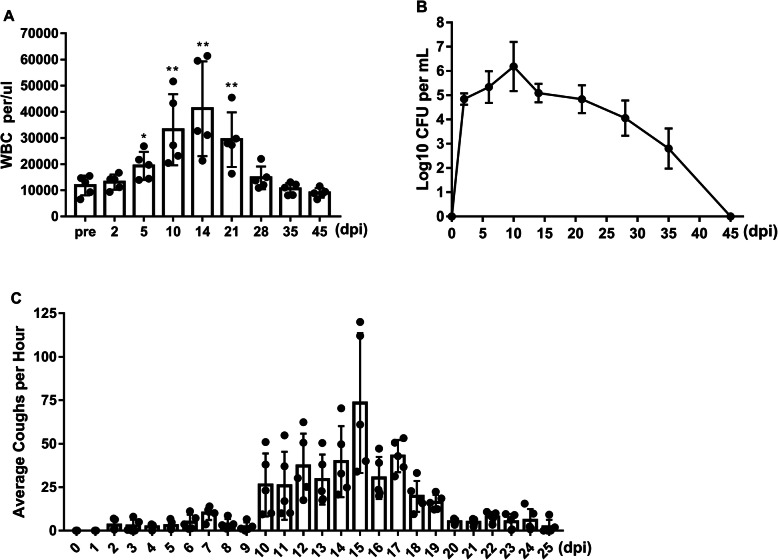
Leukocytosis, *B.p* colonization, and coughing in *B.p*-infected rhesus macaques. **a** Dynamic profiles of the number of WBCs per μL of peripheral blood in *B.p*-infected animals. **b** The CFUs per 50 μL of NPW in *B.p*-infected animals were recorded. **c** The number of coughs per day was recorded for the *B.p* s-infected group. ND, no data. **, *P* < 0.01; **, *P* < 0.005. The bars with error bars represent the means *±* SDs (*n* = 5)

The concentration of bacteria in the challenge chamber reached and was maintained at 10^4^–10^5^ CFU/mL. In the challenged group, all 5 animals developed classic symptoms of clinical pertussis. The number of WBCs was significantly increased 2- to 5-fold beginning at 6 dpi, reached the highest level at 14 dpi, and returned to baseline by 28 dpi (Fig. 2a). The number of bacterial colonies from the NPW increased from 2 dpi and reached the highest level, 6.2 × 10^6^ CFU/mL, at 10 dpi; then, the number of colonies gradually decreased until approximately 45 dpi (Fig. 2b). In addition, all animals developed severe coughs that persisted for over 4 weeks. In the early stage after challenge, the animals developed a mild cough. At 10 dpi, the cough seemed to worsen, especially at night (Fig. 2c). At peak illness, the cough became violent, lasting 10–20 s (Additional file 2). However, the rectal temperature was not significantly different from that in the pre-challenge period and was maintained between 37.2 °C and 39.9 °C (Additional file 3).

### Antibody response in the challenged group

**Fig. 3:**
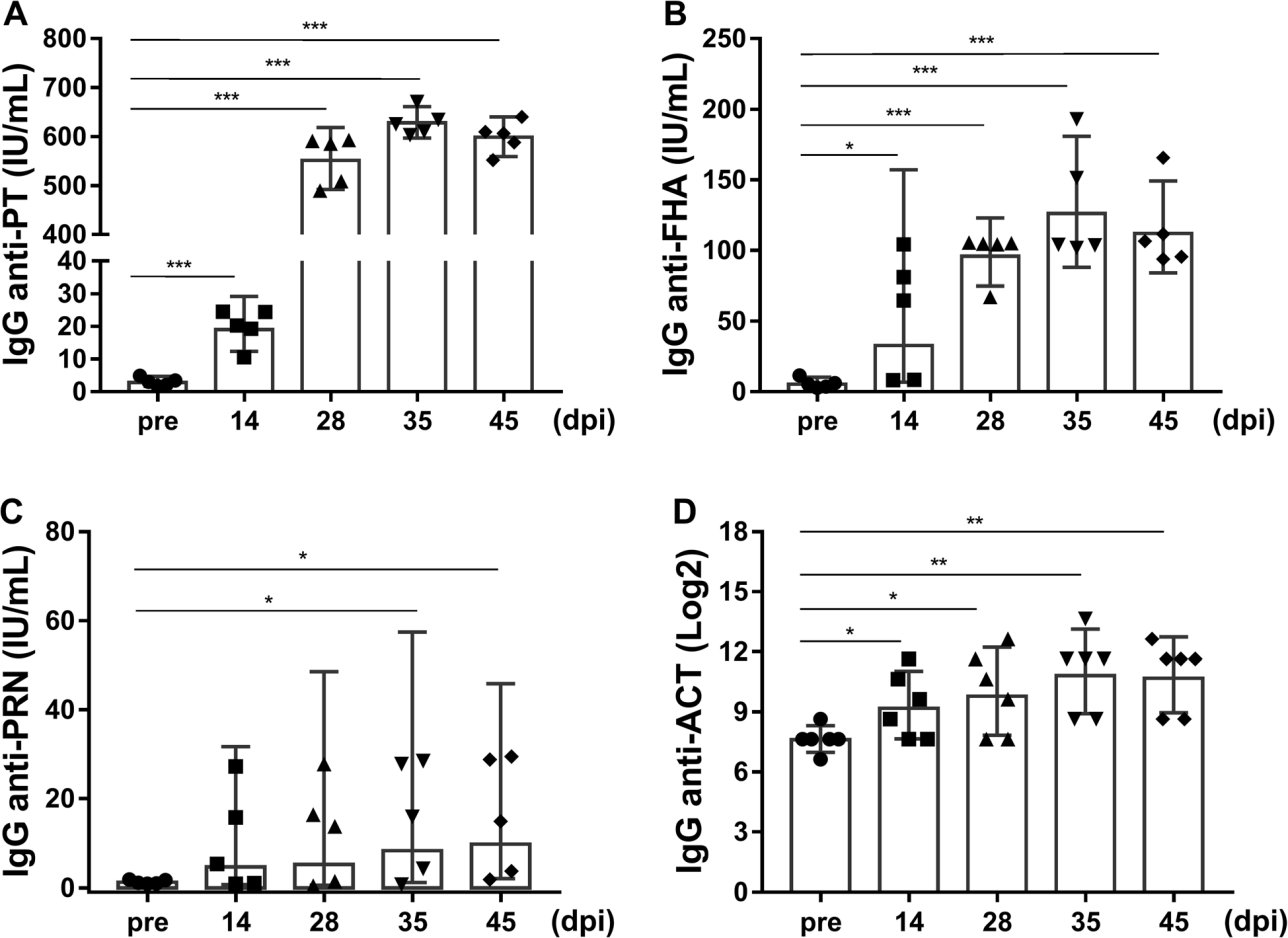
Serological responses to PT, FHA, PRN and ACT in *B.p*-infected rhesus macaques. The results are presented as GMCs or GMTs and their 95% CIs. **a** Anti-PT. **b** Anti-FHA. **c** Anti-PRN. **d** Anti-ACT. *, *P* < 0.05, **, *P* < 0.01; ***, *P* < 0.001 vs. pre-challenge; Student’s t-test. The bars with error bars represent the geometric means with 95% CIs (*n* = 5)

Production of IgG antibodies against PT, FHA, PRN, and ACT was induced in all 5 macaques in the challenged group. The seroconversion rate reached 100% at day 14 for anti-PT and anti-FHA. For anti-PRN, the seroconversion rate reached 100% at day 35 with a slight delay, but it reached 60% at day 14 and remained at 80% from day 28 onwards during the investigation period. The levels of anti-PT, anti-FHA and anti-PRN antibodies significantly increased from 14 days onwards and reached approximately 200-fold, 22-fold, and 11-fold on day 35, respectively. In addition, they remained at high levels with GMCs of 598.9 IU/mL (95% CI, 559.7–640.8), 112.0 IU/mL (95% CI, 84.11–149.2) and 9.8 IU/mL (2.08–45.88) on day 45, respectively (Fig. 3a-c). The anti-ACT levels were significantly elevated from day 14 onwards and remained stable with GMTs of 9.79 (95% CI, 7.84–12.23), 10.82 (95% CI, 8.91–13.14), and 10.68 (95% CI, 8.96–12.74), respectively, on day 28, 35, and 45 (Fig. 3c).

**Fig. 4:**
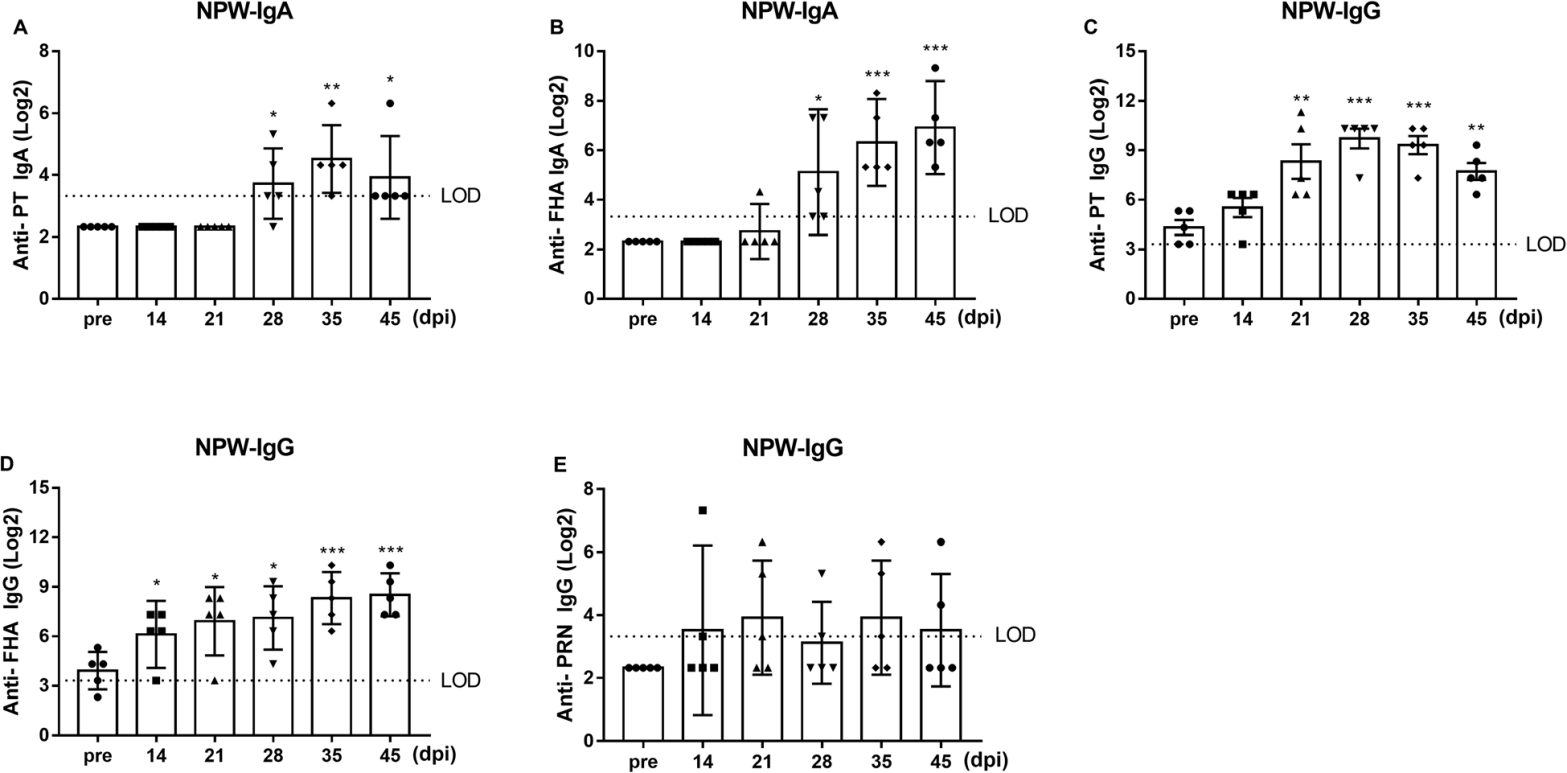
NPW antibody responses to PT, FHA, and PRN. The results are presented as GMTs and their 95% CIs. **a**-**b** Levels of IgA antibodies against PT (**a**) and FHA (**b**) in group 1 animals (*n* = 5). (C-E) Levels of IgG antibodies against PT (**c**), FHA (**d**), and PRN (**e**) in group 1 animals (*n* = 5). The dashed line represents the limit of detection (LOD, log_2_10). Values below the LOD were assigned a value of log_2_5. *, *P* < 0.05, **, *P* < 0.01; ***, *P* < 0.001 vs. pre-challenge; Student’s t-test. The bars with error bars represent the geometric means with 95% CIs

Furthermore, both IgA and IgG antibodies against PT, FHA, and PRN in NPW were assessed in all 5 animals in the challenged group at the indicated times. The levels of IgA against PT started to rise at 28 dpi and reached a maximum at 35 dpi; the levels of IgA against FHA were also significantly increased at 28 dpi and continued to rise at 45 dpi (Fig. 4a-b). However, no increases in the levels of IgA against PRN were observed in these monkeys. A previous study has suggested that human NPW contains IgG that probably enters from the circulation via transudation [19]; therefore, IgG antibodies in NPW were also evaluated. The specific IgG antibody levels in NPW at pre-challenge were very low. Anti-PT IgG levels were significantly increased in all five animals at 21 dpi and peaked at 28 dpi (Fig. 4c). Anti-FHA IgG levels rose in all five animals starting at 14 dpi and continued to increase at 45 dpi (Fig. 4d). However, not all animals showed increases in anti-PRN IgG titres after challenge (Fig. 4e).

### Transmission

**Fig. 5:**
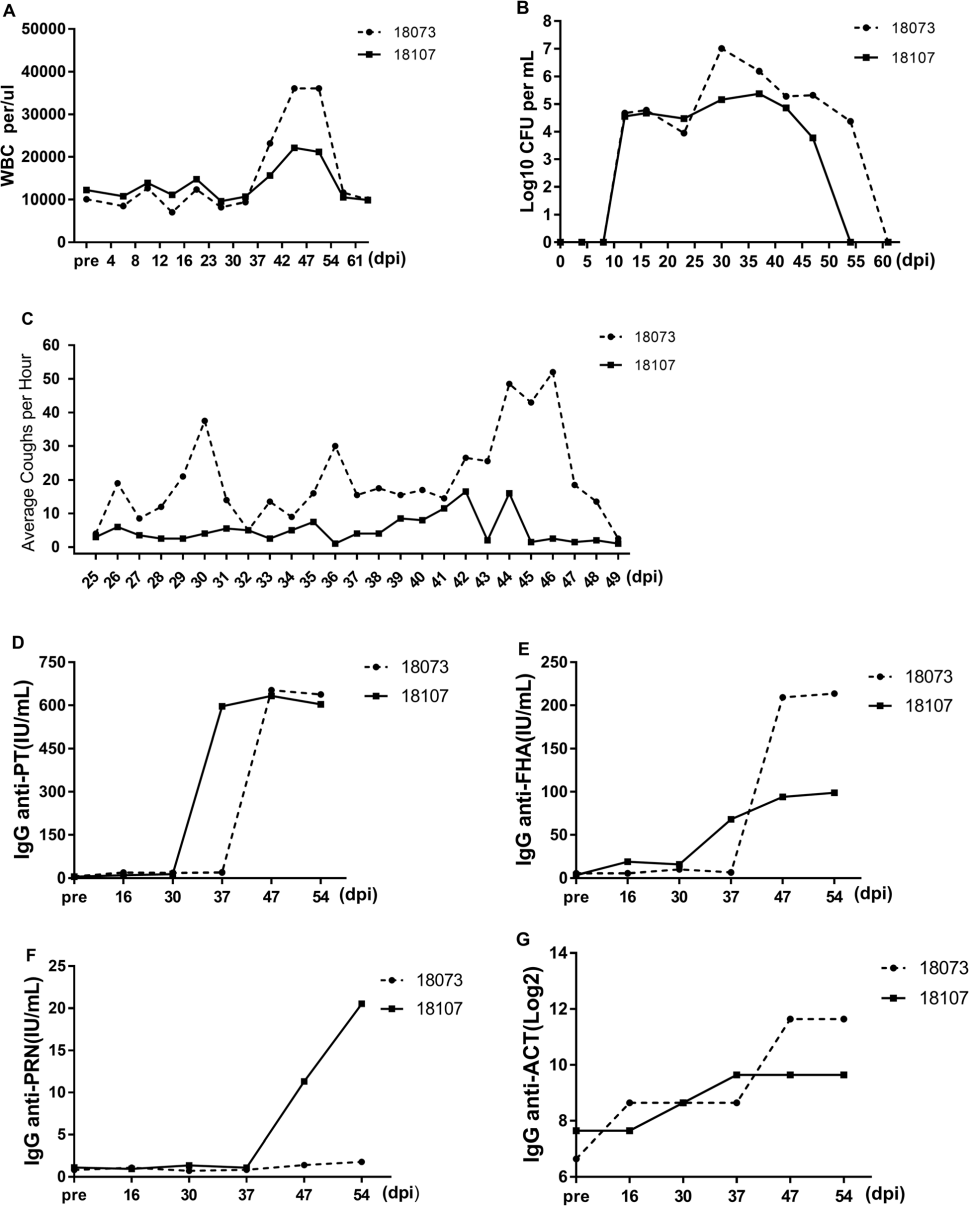
Leukocytosis, *B.p* colonization, and coughing in group 2 of *B.p*-transmitted rhesus macaques. At 2 days after challenge, 1 naive animal and 1 challenged macaque were placed in one cage for infection transmission and separated after 4 days. **a** Dynamic profiles of the number of WBCs per μL of peripheral blood. **b** The number of CFUs per 50 μl of NPW was recorded. **c** The number of coughs per day was recorded. **d**-**g** Antibody responses to the 4 *B.p* antigens (PT, FHA, PRN and ACT)

Four days after separation from the 2016-CY-41-challenged macaques, both macaques in group 2 became infected, as demonstrated by prominent leukocytosis, with a peak level between 2- and 4-fold greater than the pre-infection level (Fig. 5a). In addition, *B.p* was recovered from the NPW, with the highest numbers reaching 5.4 × 10^6^ and 7.0 × 10^6^ CFU/mL (Fig. 5b). More importantly, both animals also developed severe coughs (Fig. 5c). Antibody responses to *B.p* were also observed and exhibited trends similar to that seen following primary infection in group 1 (Fig. 5d-g).

**Fig. 6:**
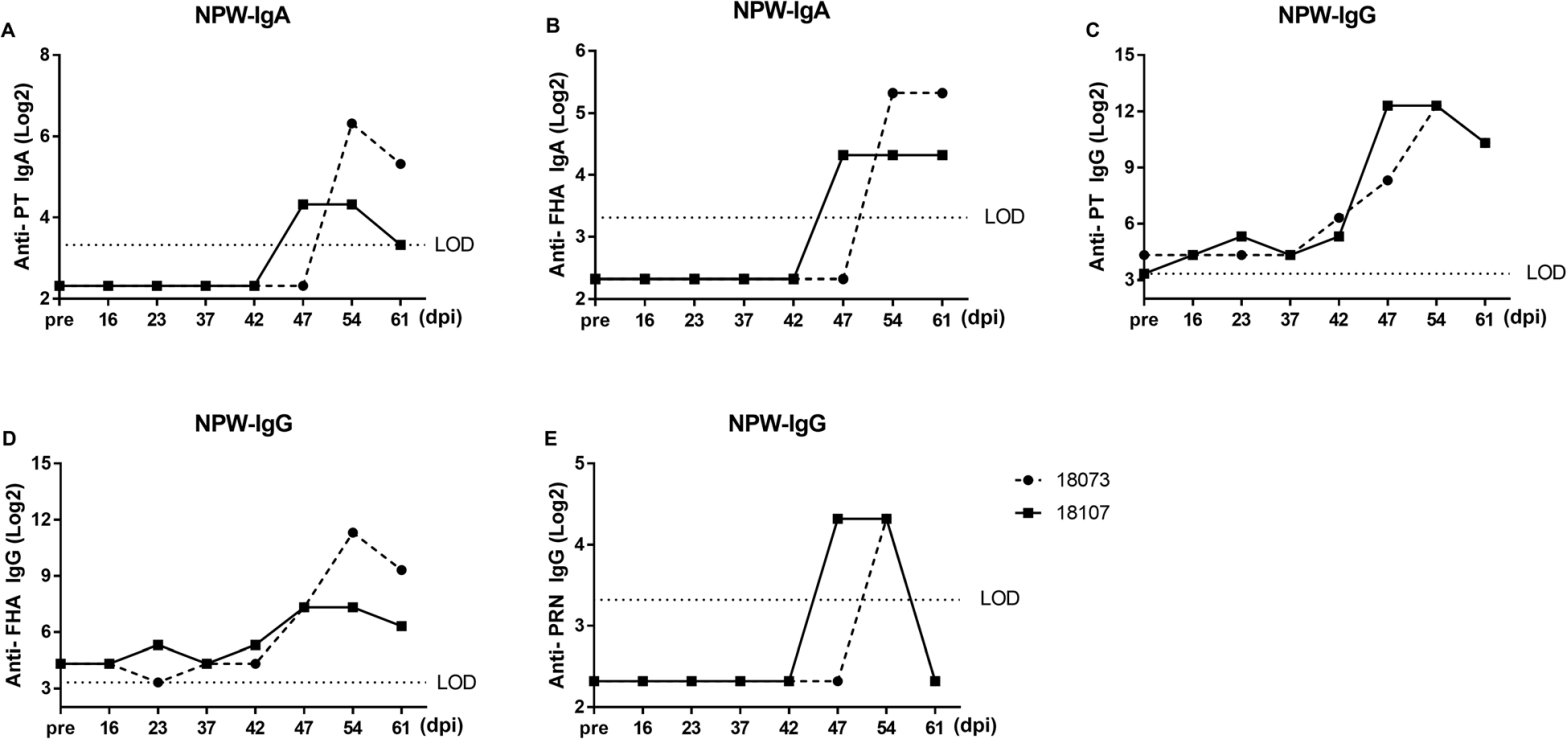
NPW antibody responses to PT, FHA, and PRN. The results are presented as the GMTs and their 95% CIs. **a**-**b** Levels of IgA antibodies against PT (**a**) and FHA (**b**) in two transmission animals. **c**-**e** Levels of IgG antibodies against PT (**c**), FHA (**d**), and PRN (**e**) in two transmission animals. The dashed line represents the limit of detection (LOD, log_2_10). Values below the detection limit were assigned a value of log_2_5. *, *P* < 0.05, **, *P* < 0.01; ***, *P* < 0.001 vs. pre-challenge; Student’s t-test. The bars with error bars represent the geometric means with 95% CIs

We also measured the levels of IgA and IgG antibodies against PT, FHA, and PRN in the NPW of the two transmission animals. IgA against PT and FHA was observed (Fig. 6a-b), and the levels of IgG against PT and FHA were also markedly increased (Fig. 6c-d). However, only a low level of anti-PRN IgG was detected for a very short period, and it declined rapidly to the pre-challenge level (Fig. 6e).

### Cytokine levels

**Fig. 7:**
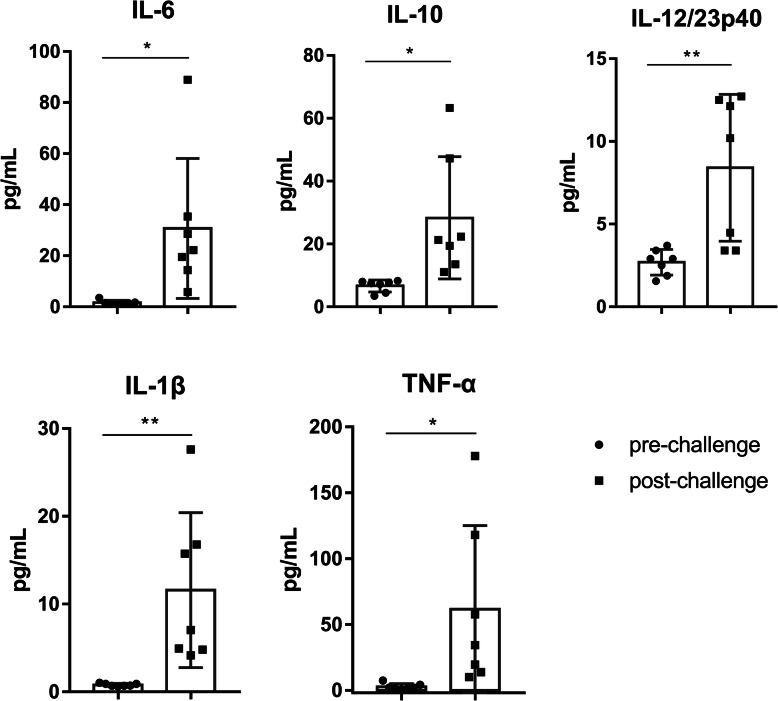
Detection of cytokines in the blood after *B. p* infection. Serum was collected from all 7 animals 1 day before challenge and 2, 6, 10, 14, 21, and 28 days post challenge for the animals in group 1 that were infected with 2016-CY-41 and 4, 8, 12, 16, 23, and 30 days post-challenge for the transmission macaques in group 2 that were cohoused with challenged animals. An unpaired t-test was used to test for differences between pre-infection serum cytokine levels and peak serum cytokine levels obtained at 2–30 dpi with *B.p* strain 2016-CY-41 since the starting concentrations were highly variable between animals, as was the day of the peak serum cytokine response. *, *P* < 0.05; **, *P* < 0.01 vs. pre-challenge; Student’s t-test. The bars with error bars represent the means ± SDs (*n* = 7)

We measured IL-1β, IL-4, IL-6, IL-8, IL-10, IL-12/23p40, IL-13, IL-17A, IFN-γ, and TNF-α in serum. The expression of the proinflammatory cytokines IL-6, IL-1β and TNF-α was significantly upregulated in all 7 macaques infected with strain 2016-CY-41(Fig. 7).

In addition, the increased IL-12/23p40 and IL-10 responses may suggest that T cell activation and regulation were increased in macaques after infection. And the time-dependent changes of these cytokines were presented in the additional file 4. However, the expression of IL-4, IL-8, IL-13, IL-17A, and IFN-γ did not exhibit significant changes.

## Discussion

Pertussis is a vaccine-preventable childhood disease; however, there has been a resurgence in cases in recent years, including in countries with good vaccine immunization rates. A deep understanding of the immunology and epidemiology of this pathogen through studies on suitable experimental models, particularly NHP models, is important. Contrary to previous rhesus macaque challenge studies, in which most animals infected with pertussis have failed to develop obvious clinical manifestations of human pertussis [10, 14, 20], the present study achieved the pertussis clinical spectrum in a rhesus macaque model. First, the characteristic whooping cough of pertussis syndrome, which has thus far been achieved only in a baboon model, was confirmed to have developed. All 5 challenged macaques and 2 macaques cohoused with the challenged macaques developed severe coughs that persisted for over 4 weeks. The coughing appeared on day 2, peaked at day 15, and decreased gradually thereafter. At peak illness, the coughing became severe, lasting 10–20 s per episode. Second, the number of CFUs in the NPW peaked at day 10 after challenge, reaching 6.2 × 10^6^ CFU/mL, and returned to baseline levels after 35 days. Third, a higher serum antibody responses of PT-IgG than of FHA-IgG was observed in serum. This is the same dynamic antibody responses as that observed in human pertussis. In humans, symptomatic pertussis is characterized by an elevated serum PT antibody response, while asymptomatic infection is characterized by an elevated serum FHA antibody response [21]. Moreover, the antibody responses in NPW also indicated that both anti-PT IgA and anti-FHA IgA levels were significantly higher than the pre-challenge levels; these changes are also observed in humans, and measurement of these antibodies has been used as a sensitive method for the diagnosis of pertussis [22, 23].

Although rhesus macaque models have been successfully established for several infectious diseases, the models for *B.p* have been insufficient [12–14]. Given the limited experimental details of the prior studies, which were performed in the 1920s–1950s, we speculated that the dose of infection, the method of infection, the age of the animal, and the infection strain may impact whether rhesus macaques can be successfully infected with *B.p.*

One of the most important factors for the pertussis model is the method of challenge. To date, nasal challenge, endotracheal intubation, in vivo injection, and aerosol challenge have been investigated in mouse models of pertussis [24, 25]. In 1929, Sauer, L.W. et al. [12] reported that typical pertussis manifestations were observed in 3 of 10 rhesus macaques after intranasal infection and in 5 of 18 Cebus monkeys after intralaryngeal infection. In 1935, Culotta, C.S. et al. [13] reported that 1 of 16 rhesus macaques were successfully infected with *B.p* via the intratracheal route, while none of five macaques were successfully infected via the intranasal route. However, the *B.p* strains and infection doses were not mentioned in these studies. We previously infected 3 rhesus macaques (4–5 years old) with strain 18,323 intranasally at a concentration of 5 × 10^8^ CFU/mL in a 2 mL volume, but none of these animals developed a cough or leukocytosis. Three animals were assigned randomly to euthanasia for scheduled necropsies at 1, 7, and 14 dpi. No pathological changes were found in the lungs, tracheas, or lung-draining lymph nodes. We observed only very low levels of bacteria in the trachea at 1 and 7 dpi, and no bacteria were observed at 14 dpi. However, no lung colonization was observed at the three scheduled time points. Recently, aerosol challenge has been used as a novel challenge method to accurately simulate natural infections and reduce animal stress [26]. Using an aerosol apparatus, we previously challenged mice with different concentrations for different periods of time and successfully established a *B.p* infection mouse model [27]. In the present study, using the aerosol apparatus, we achieved the whole spectrum of symptoms of pertussis. Compared to other methods of experimental infection, aerosol challenge has great value because it accurately simulates natural infections, exhibits superior reproducibility and results in predictable distribution of infection and pathology [28, 29].

The dose of infection should be considered during model establishment. In a recent *B.p* challenge experiment on wP-vaccinated adults, pertussis colonization exhibited dose dependence. The dose was gradually escalated from 10^3^ CFU (0% colonized) to 10^5^ CFU (80% colonized), while the minimum dose needed to induce colonization was 10^5^ CFU. In 1940, North, E.A. et al. [14] infected rhesus macaques with 2 mL of 5 × 10^7^ CFU/mL *B.p* via the intranasal route or with 1 mL of 1 mL 5 × 10^7^ CFU/mL *B.p* via the intratracheal route; however, the resultant infection was mild, and spasmodic cough and protective antibodies were not observed. Warfel, J.M. et al. [10] intranasally infected 4 rhesus macaques ranging in weight from 1.7 to 1.9 kg and ranging in age from 43 to 64 weeks with 0.5 mL of 10^9^ to 10^10^ CFU/mL *B.p*; 50% of the monkeys developed significant increases in WBC count, and one of the two monkeys with increased WBC counts developed a mild cough. However, since the infectious *B.p* strain and the animal age were not clear in North, E.A. et al.’s study, we cannot comprehensively compare our findings with those of North, E.A. et al. In the present study, infant monkeys were challenged with a recently clinically isolated strain via an aerosol apparatus in which the bacterial concentration was maintained at 10^4^–10^5^ CFU/mL for 60 min. The constant dose of *B.p* during the challenge may be one reason for the success of the present infectious pertussis model.

Another critical factor to consider when establishing an animal model is the age of the animals. A previous study using an enterovirus type 71 (EV71) rhesus macaque model showed that, of the challenged animals, a clinical spectrum similar to that of humans was observed only in young animals [30]. Moreover, studies have demonstrated that young baboons show severe disease signs, whereas adult baboons show mild signs [10]. In one baboon pertussis model, 5- to 6-week-old baboons all developed fatal pertussis; however, in juvenile baboons, the infection was not fatal [31]. Frequently, asymptomatic adults have been implicated in the spread of infection to susceptible children [32]. In our study, 5- to 6-month-old rhesus macaques were selected, and typical whooping cough, leukocytosis, bacteria-positive NPW, and transmission between animals were observed, similar to the results obtained in the baboon model. Therefore, we deduced that the challenge route as well as the age of the animals may influence *B.p* infection.

The strain of bacteria used for challenge is another factor that should be noted. Strain 18,323 was used successfully to establish pertussis in *M. cyclopis* models in the 1960s; however, it could not induce overt signs of disease in rhesus macaques [16]. A recent analysis of the global population structure of *B.p* indicated that strain 18,323 (genotype ptxP4/ptxA5/prn6/fim2–2/fim3–1) belongs to a branch containing a small number of strains that are evolutionarily far from the major prevalent branch and revealed that strain 18,323 diverged from the prevalent strain branch approximately 2000 years ago [33]. Thus, in contrast to previous macaque animal studies, our study used a strain recently isolated from a clinic in China, 2016-CY-41 (genotype ptxP1/ptxA1/prn1/fim2–1/fim3–1) (a common strain) and achieved the typical pertussis symptoms in infected macaques. Compared with that in the baboon model infected using strain D420 (genotype ptxP3/fim3–2), the peak symptom and disease progression in the macaque model infected using *B.p* strain 2016-CY-41 was delayed [10]. The prevalence of ptxP3, one of the major components of D420, has increased in many European counties, the US, and Australia in the past 25 to 30 years, rather than that of ptxp1; however, in China, ptxP1 has remained predominant [10, 34, 35]. A SNP in ptxP3 that lies in a binding site for the transcriptional regulator BvgA may result in a strong promoter and increase the level of transcription of the associated PT [36]. Strains harbouring the ptxP3 allele have been found to be more virulent than ptxP1 strains in a mouse infection model, and they may also be associated with severe disease in humans [36–38]. Thus, we hypothesize that the genomic diversity of *B.p* may affect pertussis models. Further whole-genome sequencing experiments and analyses of virulence mechanisms and pertussis epidemiology should be performed in the future.

One of the possible reasons for the previous lack of success in establishment of rhesus macaque models of pertussis is high body temperature. The results of a temperature culture test in vitro showed that ACT protein levels are significantly lower in cells grown at 39 °C than in cells grown at 37 °C, supporting the hypothesis that high temperature (39 °C) may result in loss of ACT expression, in turn resulting in a lack of *B.p* infection [10]. However, pathogenic microorganisms cultured in vitro may also exhibit virulence factor loss due to the lack of host selective pressures. During non-random culturing, *B.p* can undergo spontaneous phase variation involving multistep disappearance of virulence factors in the following order: ACT, PT and FHA [39]. The in vitro results suggest that the reduced expression of ACT is caused by the elevated normal body temperatures of rhesus macaques [10]. In the present in vivo study, we observed a 20-fold increase in the anti-ACT antibody level, which was similar to the increase observed in the baboon model [40]. In addition, the rectal temperature was between 37.2 °C and 39.9 °C and did not exhibit significant changes. Thus, we deduce that body temperature may not be the only reason why previous rhesus macaque pertussis models have failed.

## Conclusion

An infant rhesus macaque model of pertussis was established via aerosol challenge to provide a valuable alternative platform for research on pertussis pathogenesis and evaluation of vaccine candidates.

## Supplementary Information

**Additional file 1: Additional Figure 1.** Simplified layout of aerosol apparatus.

**Additional file 2. **Cough record of a rhesus macaques after aerosol challenge.

**Additional file 3: Additional Figure 2. **Rectal temperatures of rhesus macaques during the experiment.

**Additional file 4: Additional Figure 3.** Cytokines data obtained in the time-dependent manner.

## Data Availability

The datasets used and analyzed during the current study are available from the corresponding author on reasonable request.

## Abbreviations

*B.p*: *Bordetella pertussis*
NHP: Non-human primate
ptxP: Pertussis toxin promoter
ptxA: Pertussis toxin subunit
prn: Pertactin
NPW: Nasopharyngeal wash
dpi: Days post infection
WBC: White blood cell
FHA: Filamentous haemagglutinin
ACT: Adenylate cyclase toxin
