# Supplementary material for: Infant rhesus macaques as a non-human primate model of Bordetella pertussis infection
Source: BMC Infect Dis. 2021 May 3;21:407. doi: 10.1186/s12879-021-06090-y (PMC8091708; doi:10.1186/s12879-021-06090-y)
Supplement: Supplementary file 1 — Additional file 1: Additional Figure 1. Simplified layout of aerosol apparatus. [file 12879_2021_6090_MOESM1_ESM.docx]

**Additional Figure 1** **Simplified layout of aerosol apparatus.** *B. pertussis* strain 2016-CY-41 at a concentration of 10^11^ CFU/mL with 8mL were delivered to the nebulizer for aerosolisation, respectively. After aerosol generation, 10mL of air was sucked out from the sampling port every 10 min using syringes and injected into 10mL saline. The solution was transferred to Regan-Lowe charcoal agar base with 10% defibrinated sheep blood and 40 μg/mL cephalexin, and the number of colonies was determined to ensure actual challenge concentration.
