# Supplementary material for: Infant rhesus macaques as a non-human primate model of Bordetella pertussis infection
Source: BMC Infect Dis. 2021 May 3;21:407. doi: 10.1186/s12879-021-06090-y (PMC8091708; doi:10.1186/s12879-021-06090-y)
Supplement: Supplementary file 3 — Additional file 3: Additional Figure 2. Rectal temperatures of rhesus macaques during the experiment. [file 12879_2021_6090_MOESM3_ESM.docx]

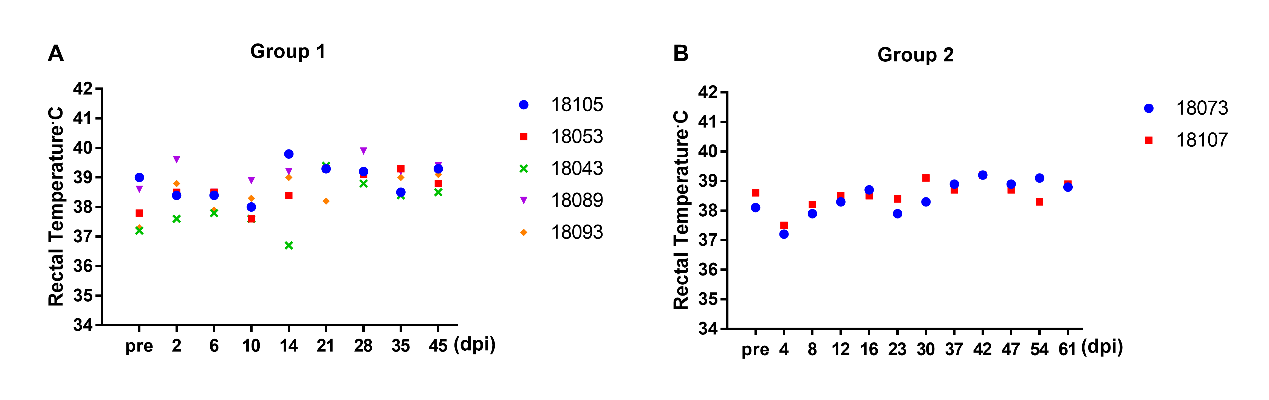


**Additional Figure 3 Rectal temperatures of rhesus macaques during the experiment.** The rectal temperature of each animal was measured at each examination postchallenge. One data point is presented for each monkey at each time point. (A) Rectal temperatures of macaques challenged with aerosolized *B.p* strain 2016-CY-41 (n=5). (B) Rectal temperatures of two animals used for strain 2016-CY-41 infection transmission.
