# Supplementary material for: Infant rhesus macaques as a non-human primate model of Bordetella pertussis infection
Source: BMC Infect Dis. 2021 May 3;21:407. doi: 10.1186/s12879-021-06090-y (PMC8091708; doi:10.1186/s12879-021-06090-y)
Supplement: Supplementary file 4 — Additional file 4: Additional Figure 3. Cytokines data obtained in the time-dependent manner. [file 12879_2021_6090_MOESM4_ESM.docx]

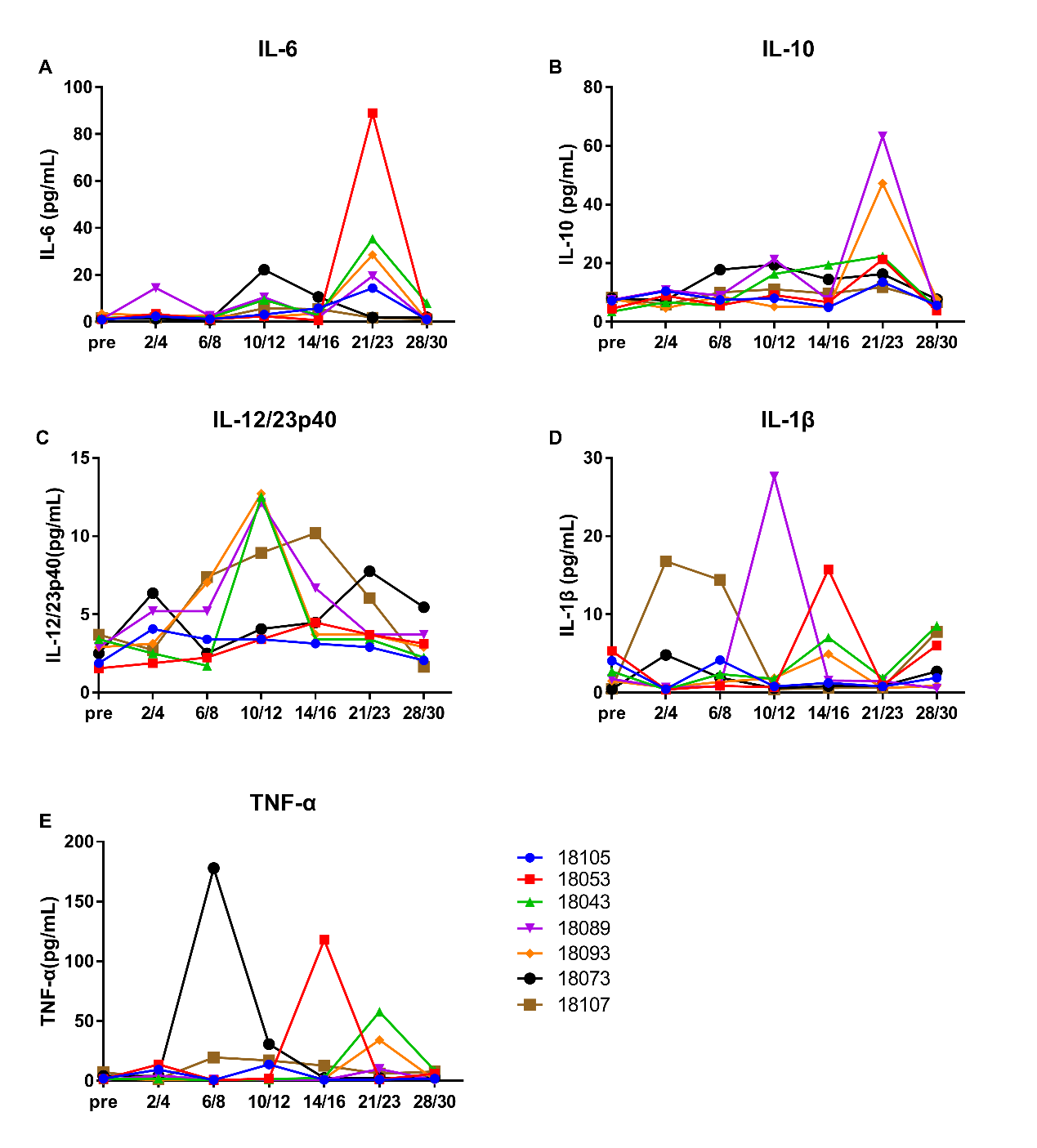


**Additional Figure 4 Cytokines data obtained in the time-dependent manner are shown.** Serum was collected from all 7 animals 1 day before challenge and 2, 6, 10, 14, 21, and 28 days post challenge for the animals in group 1 that were infected with 2016-CY-41 and 4, 8, 12, 16, 23, and 30 days post-challenge for the transmission macaques in group 2 that were cohoused with challenged animals. Levels of IL-6 (A), IL-10 (B), IL-12/23p40 (C), IL-1β (D), and TNF-α (E) were determined by the Luminex technique with a MILLIPLEX NHP Magnetic Bead Panel according to the manufacturer’s instructions. Results are presented in the time-dependent manner.
